# Musculoskeletal disorders among secondary school teachers in Douala, Cameroon: The effect of the practice of physical activities

**DOI:** 10.3389/fresc.2022.1023740

**Published:** 2022-12-16

**Authors:** Jerson Mekoulou Ndongo, Elysée Claude Bika Lele, Wiliam Richard Guessogo, Laurence Patricia Meche, Clarisse Noel Ayina Ayina, Jessica Guyot, Babette Zengue, Marie Yvonne Lobe Tanga, Léon Jules Owana Manga, Abdou Temfemo, Nathalie Barth, Bienvenu Bongue, Samuel Honoré Mandengue, Peguy Brice Assomo Ndemba

**Affiliations:** ^1^ Physical Activities and Sport Physiology and Medicine Unit, Faculty of Science, University of Douala, Douala, Cameroon; ^2^ Department of Human and Social Sciences Applied to Physical Activities and Sports, National Institute of Youth and Sports, Yaoundé, Cameroon; ^3^ Faculty of Medicine and Pharmaceutical Sciences, University of Douala, Douala, Cameroon; ^4^ INSERM, U1059, Sainbiose, Dysfonction Vasculaire et Hémostase, Université de Lyon, Université Jean Monnet, Saint-Etienne, France; ^5^ Faculty of Medicine and Biomedical Sciences, University of Yaounde I, Yaounde, Cameroon

**Keywords:** musculoskeletal disorders, prevalence, associated factors, secondary school teachers, physical activities, Cameroon

## Abstract

**Introduction:**

Musculoskeletal disorders (MSDs) represent an important threat to public health in both developed and developing countries, and are present in many occupational sectors including education. Regular practice of physical activity (PA) is known elicit preventive effects on the occurrence of MSDs.

**Objective:**

This study aimed at determining the prevalence of MSDs and the preventive impact of PA on their occurrence among secondary school teachers.

**Participants and Methods:**

A cross-sectional study was conducted among 179 teachers in five government secondary schools in Douala, Cameroon. The Nordic and Ricci-Gagnon questionnaires were used to determine MSDs and to assess the level of PA, respectively.

**Results:**

The 12-month and 7-day prevalence of MSD (PMSD-12m and PMSD-7d) were 84.3% and 69.3%, respectively. The most affected body regions by MSDs were neck (PMSD-12m = 54.2%, PMSD-7d = 33.5%), lower back (PMSD-12m = 43%, PMSD-7d = 33%), and shoulders (PMSD-12m = 35%, PMSD-7d = 22.9%). Compared to female, males were protected against MSDs during the last 12 months (OR = 0.37; 95% CI 0.16–0.93; *p* = 0.04). The risk of MSDs during the last seven days was higher in teachers aged 30-40 years (OR = 2.86; 95% CI 1.14–7.14; *p* = 0.02) and 40-50 years (OR = 4.28; 95% CI 1.49–16.29; *p* = 0.008) than those under 30 years. This risk was tripled in inactive teachers (OR = 3.07; 95% CI 1.40–6.78; *p* = 0.005), compared to their active counterparts.

**Conclusion:**

MSDs are prevalent among secondary school teachers and associated with aging, gender, and lower level of PA

## Introduction

Musculoskeletal disorders (MSDs) is a generic term encompassing a set of periarticular disorders that affect the musculoskeletal system, and mainly result in daily pain and functional discomfort (1). These also refer to gradual development of damages in musculoskeletal tissuewhich occurs when the work demand outclasses adaptive capacity of musculoskeletal tissue (2). MSDs can be localized to several body regions affecting body's joints, ligaments, tendons, nerves, muscles, and structures that support limbs, neck and back. MSDs are related to physical effort during work, thereby explaining their high occurrence in workers (3). It is also known that the origin of MSDs is multifactorial, involving a complex interplay between biomechanical stress, individual-related genetic and behavioral factors, environment factors (i.e., organizational work conditions), and psychosocial context (4, 5). These conditions are characterized by pain and limitations in mobility and functional capacity. MSDs disorders are the main contributors to disability worldwide, with low back pain being the primary global cause (6).

MSDs are a cause of public health concern in developed countries such as United States of America (USA), Canada, Finland, France, Sweden, and The United Kingdom. Indeed, MSDs are a major cause of absenteeism and inability to work (4, 6). MSDs are still largely underreported despite their enormous deleterious impact on occupational health and associated morbidity (6). In USA, MSDs accounted for 32% of all pathologies and non-fatal injuries in full-time workers in 2014 (7). Besides, MSDs are ranked first among occupational health diseases in several European countries (2).

Given the challenges related to economic growth, African countries are facing increasingly important prevalence and deleterious effects of MSDs in workers of several occupational sectors, especially in education sector. In Africa, work conditions of teachers are rude and non-ergonomic (e.g., prolonged sitting/standing, usage of inappropriate furniture, inappropriate working space), and expose them to MSDs (8, 9). Previous studies reported a high incidence of MSDs among teachers all around the world (8–12), as well as high prevalence in Africa (13, 14).

Physical activitiy (PA) is defined as any energy-requiring body movement produced by skeletal muscles (15). The World Health Organization (WHO) recommends to practice regularly at least one PA for preventing non-communicable diseases, and thus improving the quality of life (16). The benefits of regular PA on prevention of several health ailments, such as MSDs, have been largely documented (17–19). In this context, PA and sport may be efficient means for preventing, relieving, and treating MSDs (20–25).

The extensive researches on MSDs in developed countries contrasts with the lack of reports in their developing counterparts, especially in African countries, where there is a paucity of data on the prevalence and associated factors of MSDs in teachers. The effect of PA on MSD-related burden is also an important missing link in African context. In this regard, the present study aimed at determining the prevalence of MSDs and associated factors as well as the impact of PA among secondary school teachers of Douala city, Cameroon.

## Materials and methods

### Study site and population

This cross-sectional and analytical study was carried out at five Government secondary schools in the city of Douala, the economic capital of Cameroon. Teachers willing to participate in the study, with professional experience of at least one year, and having signed an informed consent form were included. Teachers with trauma history and on MSDs related medication were excluded.

### Sampling

The minimum sample size required for the study was computed using the Lorentz's formula: *N* = *p* (1-*p*) *z*^2^/*d*^2^, where *N* is the minimum sample size; *p* is the prevalence of MSDs (96%) reported previously by El Gendy and Korish (26); *z* is the statistic for the desired confidence level (*z* = 1.96 for confidence at 95%), and d is the accepted margin of error (*d* = 0.05). Thus, the minimal sample size found was *N* = 59 participants.

### Study design

**Figure 1:**
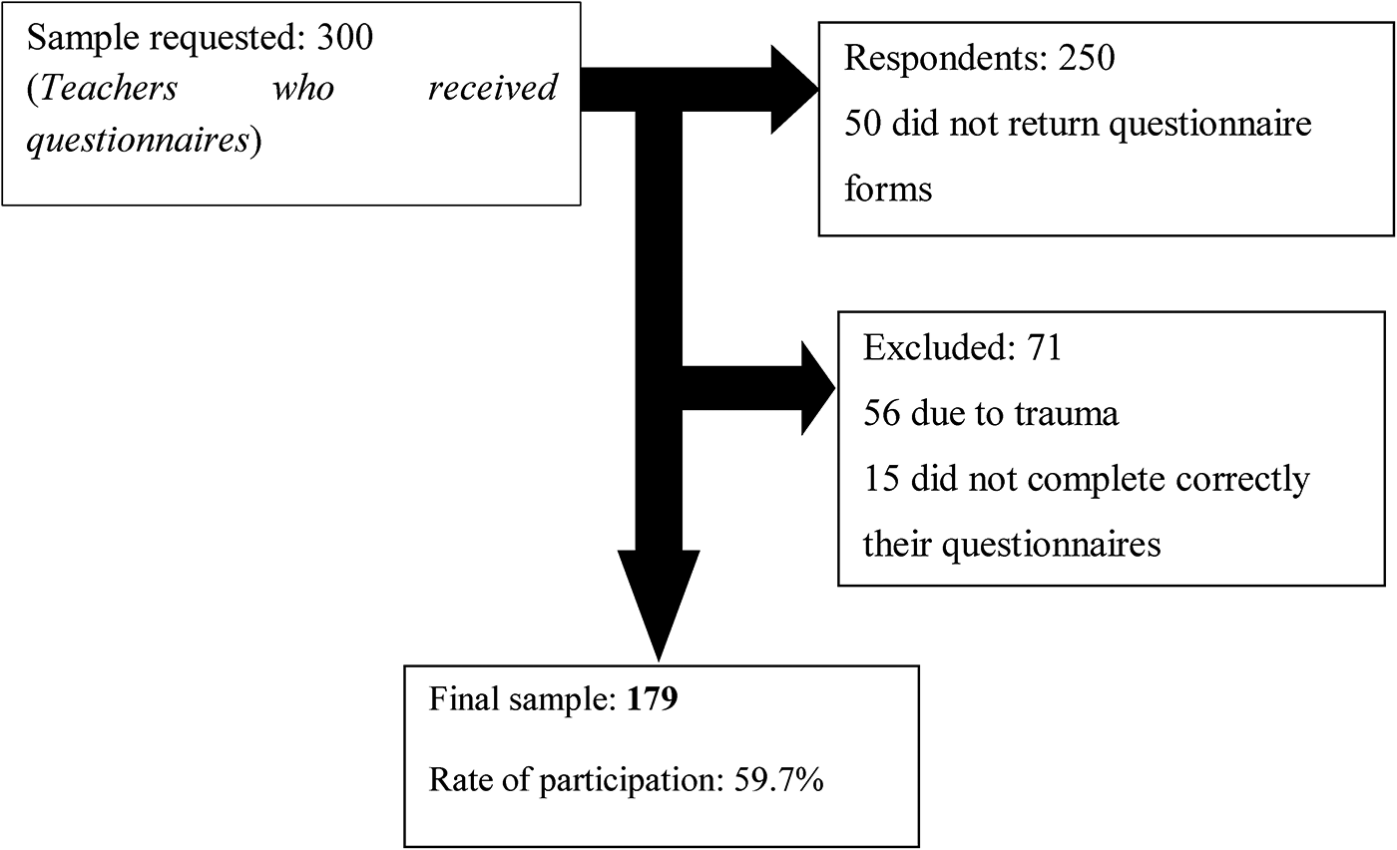
Flow charts of participants.

The study objectives and data collection methods were first explained to administration staff of the government schools, and after issuing administrative authorization, they were explained to teachers. An informed consent form was given to each teacher who accepted to participate in the study. Anthropometric parameters were measured and questionnaire forms were given to each participant. The teachers were asked to returned filled questionnaire within seven days upon reception. Teachers were assisted by research team members if questions were not well understood. Identification codes were assigned to each questionnaire to process and analyze it anonymously. Incomplete and poorly completed questionnaires were excluded from the final analysis. A total of 300 questionnaires were distributed, and 179 (60%) teachers returned correctly filled questionnaires (Figure 1).

### Data collection

A structured questionnaire was administered to each participant and consisted of three parts. (i) socio-demographic and anthropometric characteristics (age, gender, region of origin and family status weight, height), (ii) socio-professional information (school, job seniority, number of teaching hours a week); (iii) level of PA and prevalence of MSDs during the last six months or last seven days.

### Anthropometric parameters

Height was measured using a rod graduated to the nearest centimeter. Weight was measured using an electronic scale Tanita BC-532 (Tokyo, Japan). The body mass index was determined using Quetelet's formula: BMI (kg.m^−2^) = Weight (kg) / height^2^ (m^2^). Based on BMI values, participants were categorized as normal (18.5 < BMI < 25), overweight (25 ≤ BMI < 30), and Obese (BMI ≥ 30).

### Musculoskeletal disorders

The Nordic questionnaire (27) was used to determine the prevalence of MSDs. This questionnaire determines the occurrence of MSDs on nine body regions (neck, shoulders, elbows, wrists/hands, upper back, lower back, hips/thighs, knees, ankles/feet) during last 12 months or 7 days. For each body region, the following parameters were evaluated (i) the presence or absence of aches, pains or genes during the last 12 months and/or the last seven days, (ii) absenteeism or not at work during the last 12 months and/or the last seven days due to MSD in the body region concerned, and (iii) the presence or not of a history of trauma in the region concerned. Based on these three items, the prevalence of MSDs during the last 12 months (P_MSD_-_12m_) and the last seven days (P_MSD−7d_) were determined.

### Level of physical activity

The Ricci and Gagnon questionnaire was used to determine the level of PA of each participant (28). This questionnaire is a scale divided in two sub-sections, A and B, with four items each. Sub-section A evaluates the duration and intensity of daily common activities such as cleaning, gardening, rural work, and walking. Sub-section B evaluates sport and recreational activities. The total score of points in subsections A and B was used to classify participants as inactive (score < 16), active (16 ≤ score ≤ 32), and very active (score > 32).

## Statistical analysis

Qualitative variables were presented as percentages (%). Statistical analyses were conducted using the Statistical Package for Social Science v21.0 (SPSS Inc., Chicago, IL, USA). The normality of quantitative was checked using the Kolmogorov-Smirnov test. Pearson independence Chi^2^ test was used to compare proportions of unpaired samples. Logistic regression analysis was used to determine factors associated with MSD among teachers. The association between the dependent variable (presence of MSDs) and independent variables (gender, age, PA) was quantified in logistic regression analysis by computing odds ratios (OR), their confidence interval at 95%, and *p*-value. The level significance was set for a value of *p* < 0.05.

## Results

Most of the participants were females (66.5%) and aged 30–40 years (54.2%). Based on BMI analysis, 30.7% and 28.2% of teachers were overweight and obese, respectively (Table 1).

**Table 1:** Socio-demographic and professional characteristics of the participants.

Variables	Categories	*N* (%)
Age (years)	<30	27 (15.1)
	[30–40[	97 (54.2)
	[40–50[	41 (22.9)
	≥50	14 (7.8)
Gender	Male	82 (45.8)
	Female	97 (54.2)
Marital status	Divorced	2 (1.1)
	Widower	1 (1.0)
	Married	122 (68.2)
	Single	54 (30.2)
Job seniority (years)	[1–5]	48 (26.8)
	[6–9]	67 (37.4)
	[10–14]	32 (17.9)
	[15–19]	16 (8.9)
	≥20	16 (8.9)
Number of teaching hours / week	≤15 h	60 (33.5)
	>15 h	119 (66.5)
Teaching subjects	Literature/Social Sciences	96 (53.6)
	Sciences	73 (40.8)
	Physical education	10 (5.6)
Physical activities	Inactive	67 (37.4)
	Active	112 (62.6)
Body mass index	Normal	77 (43)
	Overweight	55 (30.7)
	Obese	47 (28.2)

**Figure 2:**
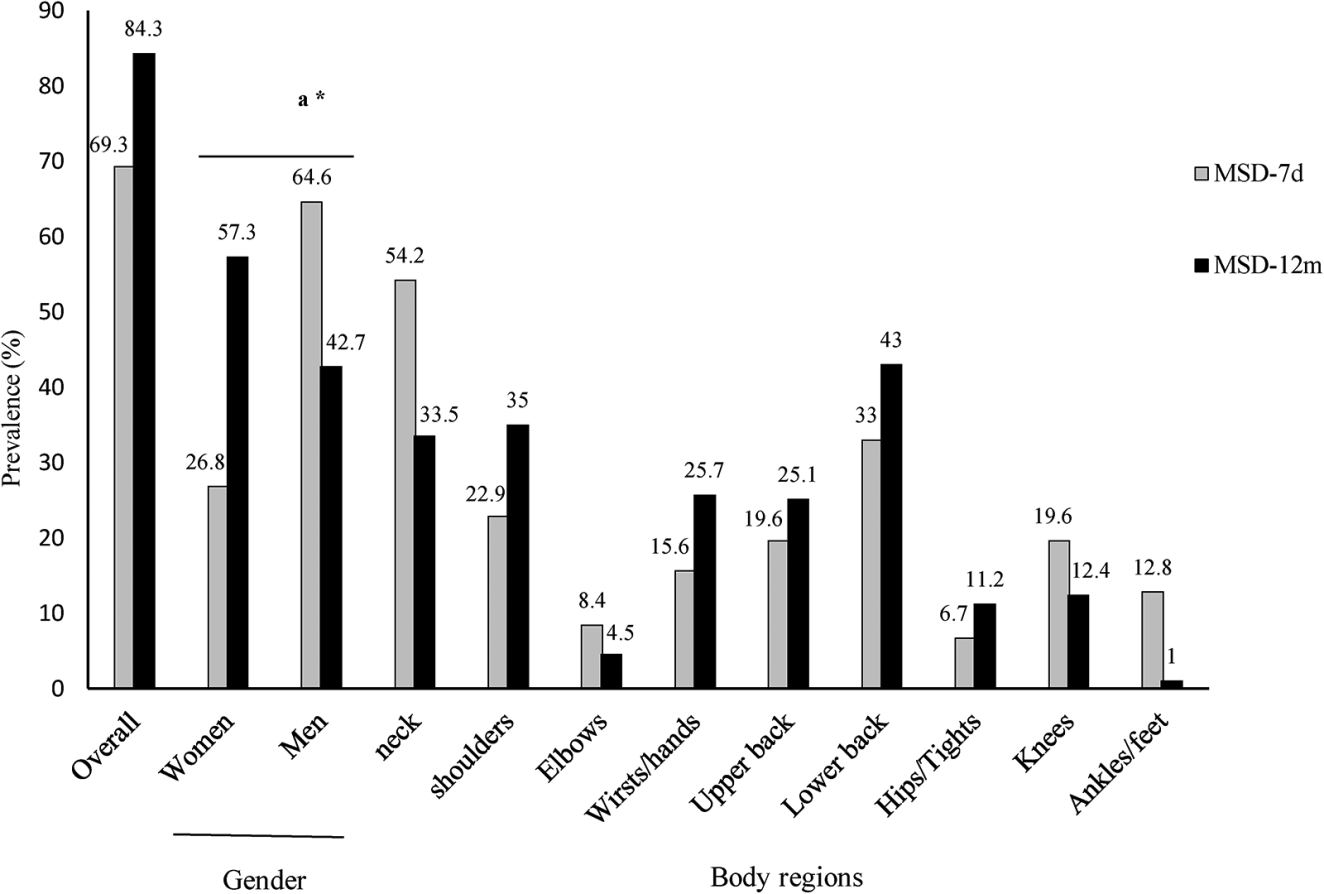
Prevalence of MSDs in body regions and gender.MSD-7d: MSD during the last 7 days; MSD-12m: MSDs during the last 12 months, *a: gender comparison of MSD-12 m between males and females*; *: *p *< 0.05.

The prevalence of MSDs by body regions and gender is depicted on Figure 2. The overall prevalence of MSD-12m and MSD-7d was 84.3% and 69.3%, respectively. P_MSD_-_12m_ value was significantly higher in females compared to males (57.3% vs. 42.7%, *p*-value = 0.03). MSD-12m and MSD-7d were most frequently reported at neck (54.2% and 33.5%), lower back (43% and 33%), and shoulders (35.2% and 22.9%).

**Figure 3:**
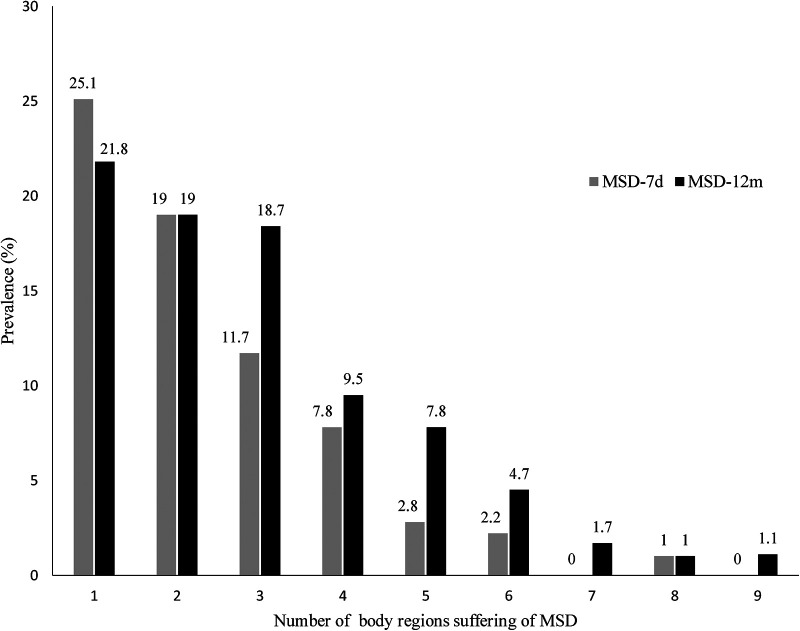
Prevalence of MSD according to the number of body regions affected. MSD-7d: MSD during the last 7 days; MSD-12m: MSDs during the last 12 months.

The teachers reported MSDs at one to nine body regions (Figure 3). The prevalence of MSD-12m and MSD-7d at one body region was 21.8% and 25.1%, respectively. Lower estimates were reported at two body regions (P_MSD_-_12m_ = 19.0% and P_MSD−7d_ = 19.0%) and three body regions (P_MSD_-_12m_ = 18.4% and P_MSD−7d_ = 11.7%) (Figure 3).

On multivariate regression analysis, the risk of MSDs was increased by ∼three times in teachers aged 30–40 years (OR = 2.86; 95% CI 1.14–7.14; *p* = 0.02), and by over four times in those aged 40–50 years (OR = 4.28; 95% CI 1.49–16.29; *p *= 0.008), compared to those aged below 30 years old. It was also noticed positive effect of PA on the risk of MSD-7d. Teachers classified as “inactive” were three times at risk of MSDs (OR = 3.07; 95% CI 1.40–6.78; *p* = 0.005), compared with those classified as “active”. In contrast, the risk of MSD-12 m was decreased by 63% in males (OR = 0.37; 95% CI 0.16–0.93; *p* = 0.04) (Table 2).

**Table 2:** Multivariate logistic analysis of associated factors with MSDs.

	Parameters	Categories	MSDs + (%)	OR (95%CI)	*p*-value
	Gender	Female	57.30	1	
		Male	42.70	0.37 (0.16–0.93)	**0** **.** **04**
MSD-12m	Gender	Female	58.01	1	** **
		Male	41.94	0.40 (0.19–0.88)	**0** **.** **02**
MSD-7 d	Age (years)	<30	48.15	1	
		[30–40[	72.16	2.86 (1.14–7.14)	**0** **.** **02**
		[40–50[	70.05	4.28 (1.49–16.29)	**0** **.** **008**
		≥50	62.29	1.90 (0.44–8.28)	0.39
	Physical activities	Active	62.50	1	
		Inactive	80.60	3.07 (1.40–6.78)	**0** **.** **005**

MSDs, Musculoskeletal disorders.

MSD-12m, MSDs during the last 12 months; MSD-7d, MSDs during the last 7 days.

## Discussion

The purpose of this study was to assess the prevalence and determinants of MSDs as well as the impact of PA on MSDs risk, among secondary school teachers of Douala, Cameroon.

A P_MSD_-_12m_ of 84.3% was in this study, thereby indicating that MSDs were very frequent among secondary teachers. This prevalence is consistent with that reported in different settings including Botswana (83.3%), Nigeria (70.2%), Saudi Arabia (68.50-79.17%), and Philippines (79.17%) (14, 29, 30). Higher P_MSD_-_12m_ estimates were reported in Chile (90.81%) (31) and Egypt (96%) (26).

More than half (69.3%) of teachers suffered from MSDs during the last seven days, and this is in line with that reported in Bolivia (63.4%) by Solis-Soto et al. (32). Facing execrable working conditions daily e.g., prolonged sitting/standing could explain this high P_MSD−7d_ estimate reported here (8, 9). Several authors opined a multifactorial origin of MSDs including prolonged static muscle load and repetitive work gestures (33–36). A higher teaching load could also explain this high MSDs prevalence. It was reported a teacher-to-student ratio of 1/377 in Cameroon, which is 15 times higher than the UNESCO recommended 1/25 ratio (37, 38).

Consisting with previous studies (26, 39), MSDs were mainly located at neck. In contrast, it was reported a predominance of MSDs at shoulders in Indian and Nigerian teachers, while MSDs were mainly seen at lower back of Kenyan teachers (14, 40, 41). Differences related to working environment and teaching approaches could likely explain these discrepancies observed between the above mentioned studies and the present study. The increased risk of MSDs among older participants found in this study was also reported earlier in other settings (10, 39, 40).

P_MSD_-_12m_ was significantly higher in women compared to men. Similar observations were done elsewhere on an increased risk of MSD-12m at various body parts (neck, shoulders, upper back, and feet/knees) in female gender in the educational sector (11, 29, 42). According to Ng et al. (43), this predisposition of women to MSDs is due to the fact they are predominant in teaching profession as it was the case in our study. Chong and Chan (9) suggested that females might suffer more frequently from MSDs than males because of lower physical strength, and higher difficulties to manage relatives and professions-elicited stress.

This study confirms the protective role of PA against MSDs, and this finding was also reported in diverse studies on education sector (25, 29, 44, 45), and other occupational sectors (21, 22, 23, 25, 46). Again, other studies found the positive role of PA in improving MSDs symptoms in all body regions (17, 18, 19). Erick and Smith (29) pointed out that PA ≥ 5 h/week was protective against MSDs. On a physiological view, PA increases blood supply to muscles and bones, reduces muscle tension, and preserves joint movement, minimizing pain, injuries, and enhancing their repair (44, 47). Moreover, sufficient PA will result into reduced pain, strengthened weak muscles, and decreased mechanical load on vertebral structures (48). It has been suggested that a regular exercise of ≥30 min/day could stimulate an adequate production of endorphins which are known to reduce MSDs (49, 50).

### Limitations

Causes of MSDs being multifactorial, some of them constitute the limits of this study. Therefore, crucial determinants such as psychosocial factors, job/salary satisfaction, quality of life, exposure to workplace violence, and ergonomic factors were not captured, and this represents an important limitation to this study. Also, medical confirmation of MSDs diagnostic was not done. Thus, MSDs prevalence estimates based on the Nordic questionnaire could not reflect the real burden of MSDs among teachers.

## Conclusion

This study found a high prevalence of MSDs among Cameroonian secondary school teachers, with a predominance of MSDs at neck, lower back, and shoulders. The study also outlined protective effect of PA in reducing risk of MSDs. It is critical to implement primary MSDs prevention measures to preserve wellbeing of teachers through improvement of work conditions and promotion of PA and sport. All taken together could be very helpful to prevent and control efficiently education sector-related MSDs.

## Data Availability

The raw data supporting the conclusions of this article will be made available by the authors, without undue reservation.
